# Long-Term Outcomes and Paired Immune and Genomic Exploratory Analyses After Neoadjuvant Dose-Dense MVAC in Muscle-Invasive Bladder Cancer: A Single-Centre Case Series

**DOI:** 10.3390/cancers18172839

**Published:** 2026-09-02

**Authors:** Daan De Maeseneer, Alexander Decruyenaere, Joni Van der Meulen, Siebe Loontiens, Toon Rosseel, Pieter-Jan Volders, Sofie Verbeke, Valerie Fonteyne, Charles Van Praet, Camille Berquin, Sylvie Rottey

**Affiliations:** 1 Department of Medical Oncology, Ghent University Hospital, 9000 Ghent, Belgiumsylvie.rottey@uzgent.be (S.R.); 2 Department of Medical Oncology, AZ Sint-Lucas, 8310 Brugge, Belgium; 3 Molecular Diagnostics, Ghent University Hospital, 9000 Ghent, Belgium; joni.vandermeulen@uzgent.be (J.V.d.M.); siebe.loontiens@ugent.be (S.L.); 4 Department of Biomolecular Medicine, Ghent University, 9000 Ghent, Belgium; toon.rosseel@ugent.be (T.R.); pieterjan.volders@ugent.be (P.-J.V.); 5 Department of Pathology, Ghent University Hospital, 9000 Ghent, Belgium; sofie.verbeke@uzgent.be; 6 Department of Radiotherapy-Oncology, Ghent University Hospital, 9000 Ghent, Belgium; valerie.fonteyne@uzgent.be; 7 Department of Urology, Ghent University Hospital, 9000 Ghent, Belgiumcamille.berquin@uzgent.be (C.B.); 8 Department of Human Structure and Repair, Faculty of Medicine and Health Sciences, Ghent University, 9000 Ghent, Belgium

**Keywords:** muscle-invasive bladder cancer, neoadjuvant chemotherapy, dose-dense MVAC, pathologic complete response, chemoresistance, tumour mutational burden, tumour immune microenvironment, treatment sequencing

## Abstract

**Background/Objectives**: Cisplatin-based neoadjuvant chemotherapy (NAC) followed by radical cystectomy has been the standard of care for muscle-invasive bladder cancer (MIBC) for two decades, yet a substantial proportion of patients derive no benefit. As antibody–drug conjugates and immune checkpoint inhibitors reshape perioperative treatment, it is unclear which patients retain meaningful benefit from platinum. We describe long-term outcomes and exploratory paired immune and genomic analyses in a single-centre cohort treated with dose-dense MVAC (dd-MVAC). **Methods**: We retrospectively identified 54 consecutive patients with MIBC treated with neoadjuvant dd-MVAC between November 2013 and November 2019. Forty-two underwent radical cystectomy and are assessable for pathologic response. Immunohistochemistry for CD3, CD8, FOXP3, PD-1, PD-L1, PD-L2, and NY-ESO-1 was evaluable in 31 baseline specimens and 22 paired specimens; paired genomic profiling was evaluable in 12 cases, with paired tumour mutational burden (TMB) in 10 by whole-exome sequencing and 7 by TSO-500. Paired analyses necessarily exclude patients achieving a pathologic complete response (pCR), who have no residual tumour, and most early progressors. **Results:** pCR was achieved in 11/42 operated patients (26%, 95% CI 15–41). Median follow-up was 87 months (IQR 24–104). Hydronephrosis was the only baseline factor associated with absence of pCR (*p* = 0.016). Within the operated cohort, pCR was associated with longer recurrence-free survival (log-rank *p* = 0.017), but the difference in overall survival did not reach significance (*p* = 0.121). NAC reduced intratumoral FOXP3 (q = 0.001), NY-ESO-1 (q = 0.013), PD-L2 (q = 0.013), and CD3 (q = 0.043) after correction for multiple comparisons, whereas TMB showed no significant change (TSO-500 *p* = 0.67; WES *p* = 0.86). No statistically significant association was detected between any baseline immune marker, including PD-L1, and pCR. The mutational landscape was largely concordant before and after NAC. **Conclusions:** This study was exploratory and was not powered to validate predictive biomarkers. In this long-term cohort, dd-MVAC was associated with measurable depletion of intratumoral immune populations in chemoresistant tumours, while paired genomic profiles remained broadly concordant. No pre-treatment biomarker identified platinum-refractory patients, but the small evaluable subsets mean these are hypothesis-generating rather than negative findings, and prospective studies with pre-specified biomarker endpoints are required.

## Simple Summary

Bladder cancer that has grown into the muscle of the bladder wall is treated with chemotherapy followed by surgical removal of the bladder in large parts of the world. This chemotherapy works well in some patients but not in others, and it is not yet possible to predict in advance who will benefit. In this study, we followed 54 patients who received intensive chemotherapy before surgery and were monitored for a median of more than seven years. We examined features of each tumour before and after chemotherapy, including the genes that were altered, the number of mutations in the tumour, and the immune cells present within it. We found that chemotherapy reduced several immune cell populations in the tumour but did not change the underlying genetic make-up or the overall mutation count. None of the markers measured before treatment reliably identified patients who failed to respond. These findings may help explain why chemotherapy fails in some patients and inform how newer drugs might best be sequenced.

## 1. Introduction

Bladder and urinary tract urothelial cancer (UC) is a major health burden. In 2022, an estimated 614,000 new cases and 221,000 deaths were recorded worldwide, with approximately three-quarters of cases occurring in men and the highest incidence rates reported in Europe, with an age-standardised incidence of 20–29 per 100,000 [1].

The systemic treatment landscape has evolved drastically with the introduction of immune checkpoint inhibitors (ICI) and antibody–drug conjugates (ADC) into international guidelines [2,3]. Although of considerable historical value, the place of platinum-based chemotherapy (PBC) has become uncertain, and the risk–benefit ratio of cisplatin or carboplatin is increasingly deemed unfavourable by both physicians and patients. In muscle-invasive bladder cancer (MIBC), neoadjuvant PBC was long the mainstay of treatment because of a clear overall survival (OS) benefit and a meaningful rate of pathologic complete response (pCR) [4,5,6].

This paradigm is now being directly challenged. The phase 3 KEYNOTE-B15/EV-304 trial, combining the ICI pembrolizumab with the ADC enfortumab vedotin (EV), outperformed PBC in cisplatin-eligible patients in event-free survival, OS, and overall response. Compared with cisplatin–gemcitabine, the pCR rate was significantly higher for EV–pembrolizumab (55.8% vs. 32.5%; one-sided *p* < 0.0001) [7]. However, because the control arm received neither perioperative ICI (durvalumab, NIAGARA trial [8]), adjuvant ICI (nivolumab in CheckMate 274 [9]; pembrolizumab in AMBASSADOR [10]; atezolizumab in IMvigor011 [11]), nor a more effective PBC regimen such as dose-dense MVAC (dd-MVAC, VESPER trial [6]), the magnitude of this benefit may be overestimated. Ongoing randomised trials will more clearly define the residual value of PBC in the peri-operative setting.

In locally advanced and metastatic UC (la/mUC), PBC remains relevant from the second line onward, particularly in patients without *FGFR* alterations. The EV-302 trial demonstrated a clear first-line benefit of EV–pembrolizumab over PBC (median OS 31.5 vs. 16.1 months) [12]. In HER2-expressing la/mUC, disitamab vedotin combined with toripalimab has likewise outperformed PBC in the first line (median OS 31.5 vs. 16.9 months) [13], and trastuzumab deruxtecan has shown activity in HER2-expressing tumours [14]. Nevertheless, most patients ultimately progress on these therapies and are treated with PBC in second or later lines: in the EV-302 trial, 85.9% of patients in the EV–pembrolizumab arm who received any subsequent systemic therapy received PBC, and in the RC48-C016 trial [13] 74.2% of patients in the disitamab vedotin group did so.

Two questions therefore remain open. First, it is not known whether the immune and genomic features of a tumour before treatment can serve as predictive markers of failure to respond to platinum—a question distinct from the well-established prognostic value of pathologic response itself. Second, it is not known how a completed course of intensive platinum chemotherapy alters the tumour immune microenvironment and genome, which bears directly on whether checkpoint inhibition should be given concurrently with, or sequentially after, chemotherapy. Paired pre- and post-treatment tissue from a uniformly dd-MVAC-treated cohort is an uncommon resource with which to address the second question and remains informative even as newer regimens emerge.

In this article, we therefore describe, in 54 consecutive patients treated with neoadjuvant dd-MVAC and followed for a median of more than seven years: long-term outcomes by pathologic response; the association between baseline immune and genomic features and response; and paired changes in the immune microenvironment, tumour mutational burden, and mutational landscape after chemotherapy. All biomarker analyses are exploratory and hypothesis-generating; the study was not designed or powered to validate a predictive biomarker.

## 2. Materials and Methods

### 2.1. Patient Selection and Data Extraction

**Figure 1:**
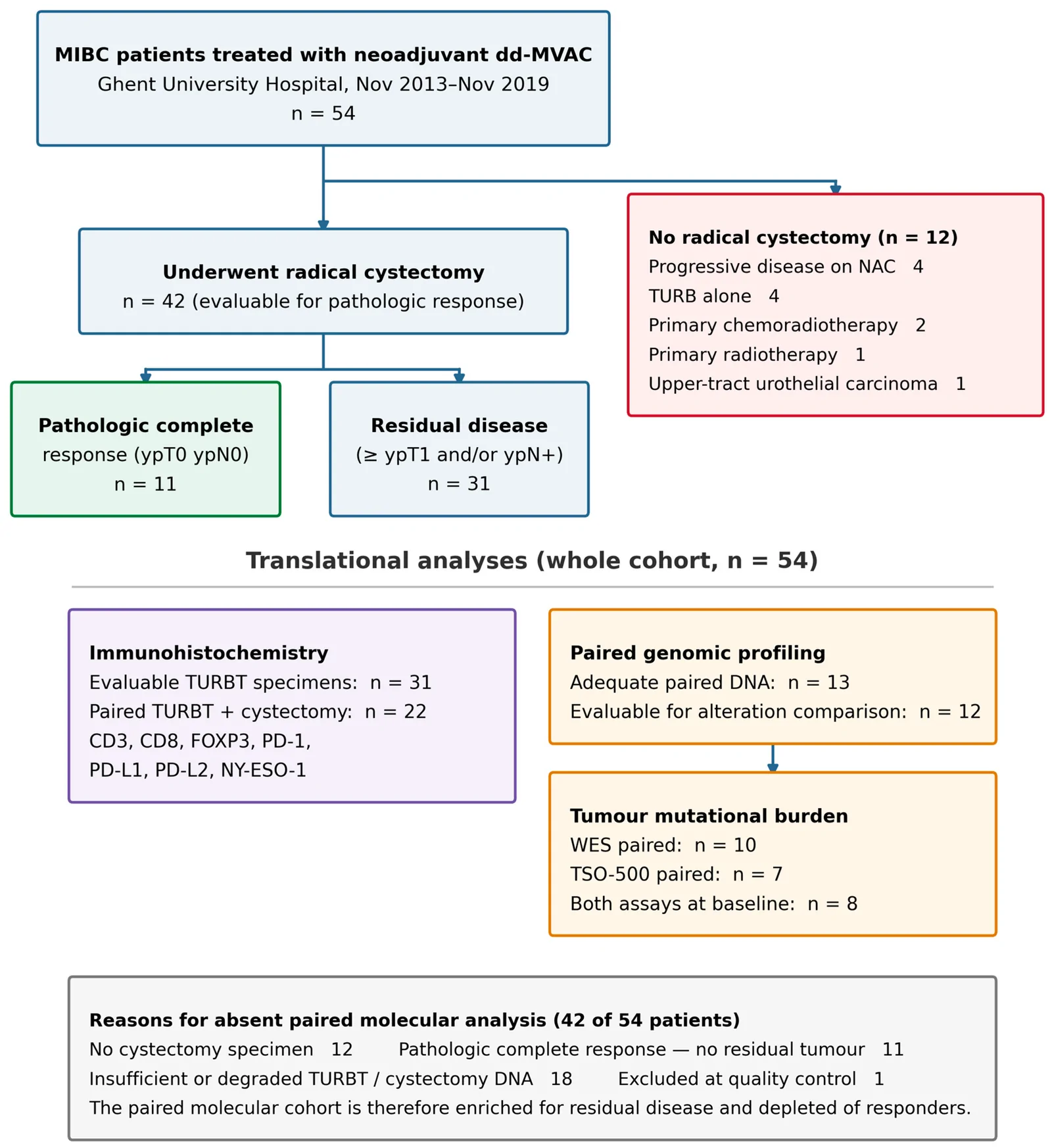
Flow of patients through the study. Of 54 consecutive patients treated with neoadjuvant dd-MVAC, 42 underwent radical cystectomy and were evaluable for pathologic response. Paired translational analyses were possible in a minority of patients; the reasons for absent paired molecular analysis are shown and make clear that the molecular subset is enriched for residual disease and depleted of complete responders. NAC, neoadjuvant chemotherapy; TURB(T), transurethral resection (of bladder tumour); WES, whole-exome sequencing.

After review and authorisation by the local ethics committee (Ghent University Hospital, reference 20171080), we identified 54 consecutive patients with muscle-invasive bladder cancer who received dose-dense MVAC in the neoadjuvant setting at Ghent University Hospital between November 2013 and November 2019. Patient characteristics and follow-up data were extracted from the electronic patient file and anonymised before analysis. The flow of patients through the study, including all reasons for non-contribution to each analysis, is shown in Figure 1.

Eligible patients had histologically confirmed urothelial carcinoma with muscle invasion (≥cT2) on transurethral resection of the bladder tumour (TURBT), no radiological evidence of distant metastasis and fitness for cisplatin-based chemotherapy. Cisplatin eligibility was assessed according to the Galsky criteria [15] and reviewed at the multidisciplinary uro-oncology board before treatment; all patients had a creatinine clearance ≥ 60 mL/min. Patients treated with carboplatin-containing or gemcitabine-based regimens, and those given dd-MVAC in the adjuvant or metastatic setting, were excluded. Clinical staging was based on magnetic resonance imaging of the pelvis with computed tomography of the thorax and abdomen.

Chemotherapy consisted of four planned cycles of dd-MVAC at 14-day intervals with granulocyte colony-stimulating factor support, followed by radical cystectomy with bilateral pelvic lymph-node dissection approximately four to six weeks after the last cycle. Adjuvant radiotherapy was considered at the multidisciplinary board for patients with adverse pathology after cystectomy, defined as positive surgical margins, extravesical extension (≥ypT3) or node-positive disease.

Pathologic complete response (pCR) was defined as the absence of residual invasive tumour and nodal involvement in the cystectomy specimen (ypT0 ypN0), and residual disease as any residual invasive carcinoma (≥ypT1) and/or nodal involvement. Patients who did not undergo cystectomy have no pathologic-response status and are analysed as a separate descriptive group throughout.

### 2.2. PD-L1 Determination

Pathology specimens from the diagnostic TURBT and from the radical cystectomy were stained for PD-L1 using two monoclonal antibodies: clone 22C3 (PD-L1 IHC 22C3 pharmDx, Dako/Agilent, Santa Clara, CA, USA) and clone SP142 (VENTANA PD-L1 [SP142] Assay, Roche Diagnostics, Tucson, AZ, USA). Staining was performed on 4-µm formalin-fixed, paraffin-embedded sections according to the manufacturer’s protocol on the VENTANA BenchMark ULTRA platform; cross-platform use of Dako antibodies on the BenchMark ULTRA has been validated and shows interchangeable performance with the corresponding Dako Autostainer Link 48 (Dako Denmark A/S, Glostrup, Denmark) [16]. For both assays, PD-L1 positivity was defined as ≥1% of viable tumour cells showing membranous staining of any intensity (tumour-cell [TC] score ≥ 1%). A uniform tumour-cell-based threshold was applied to both antibodies to allow direct head-to-head comparison; the conventional companion-diagnostic cut-offs (combined positive score ≥ 10 for 22C3 and immune-cell score ≥ 5% for SP142) were recorded as secondary categories. A combined variable (PD-L1 positive if either antibody and/or timepoint was positive) was used for the primary analysis. We note that the SP142 clone is known to show a distinctly different staining pattern from the 22C3, 28-8, and SP263 clones [17].

### 2.3. Immunohistochemistry and Antibody Staining

Immunohistochemistry was performed at the Department of Pathology, Ghent University Hospital, on formalin-fixed paraffin-embedded tissue from the diagnostic TURBT and, where available, from the radical cystectomy specimen. Sequential 4-µm sections were cut from the same paraffin block for each time point so that all markers were assessed on immediately adjacent tissue. Heat-induced epitope retrieval was performed with cell-conditioning solution 1 (Tris–EDTA-based, pH 8.5) for all markers except NY-ESO-1, for which cell-conditioning solution 2 (citrate-based, pH 6) was used. Staining was carried out on the VENTANA BenchMark ULTRA automated platform (Roche Diagnostics, Tucson, AZ, USA) with endogenous peroxidase blocking, incubation with the primary antibody, and diaminobenzidine (DAB) detection according to the manufacturer’s instructions. Slides were counterstained with haematoxylin.

The panel comprised CD3 (clone 2GV6), CD8 (C8/144B), FOXP3 (236A/E7), PD-1 (NAT105), PD-L1 (22C3 and SP142), PD-L2 (366C.9E5), and NY-ESO-1 (E978); clones, suppliers, dilutions, and retrieval conditions are listed in Supplementary Table S1. Every staining run included an on-slide positive control (tonsil for the immune markers, testis for NY-ESO-1) and a negative control in which the primary antibody was omitted. Runs in which the control tissue failed to stain as expected were repeated. Cross-platform use of Agilent (Dako) antibodies on the VENTANA BenchMark ULTRA has been validated and shows performance interchangeable with the corresponding Dako Autostainer Link 48 [16].

Immune infiltration was assessed semi-quantitatively by categorical visual estimation of infiltrate density rather than by cell counting or digital image analysis, following the approach we have applied previously in oropharyngeal squamous cell carcinoma [18,19]. For each case, a haematoxylin-and-eosin slide was reviewed first to confirm invasive urothelial carcinoma and to delineate the areas suitable for scoring; necrotic, ulcerated and cauterised areas were excluded, which is particularly relevant in TURBT material. Three randomly chosen fields at ×20 objective magnification were examined per slide, and an overall four-level grade was assigned for each marker and specimen: none, low, moderate or strong, coded 0–3 for analysis. CD3, CD8, FOXP3, and NY-ESO-1 were graded on this scale. PD-1 and PD-L2 were recorded as the percentage of positive cells and PD-L1 as described in Section 2.2. Staining of any intensity was counted as positive, because intensity is sensitive to pre-analytical variables that could not be controlled retrospectively.

All slides were scored independently by a uropathologist (S.V.) and the first author (D.D.M.), both blinded to pathologic response, clinical outcome, and specimen timepoint. Discordant scores were re-reviewed jointly and resolved by consensus, and the consensus score was used in all analyses. Criteria for classifying a specimen as impossible to evaluate, together with the composite numerical score used in our earlier work and the corresponding sensitivity analysis, are detailed in Supplementary Methods S2 and Supplementary Table S6.

### 2.4. Genetic Analysis

Paired genetic analysis was attempted in all patients with adequate residual material. Thirteen patients had adequate paired DNA, 12 were evaluable for the alteration-level comparison, and 10 were fully evaluable at both timepoints. Reasons for non-contribution are given in Figure 1. The constraint is structural rather than random: patients achieving a pCR have no residual tumour to sequence, so the paired genomic cohort is by construction enriched for chemoresistant disease.

DNA was extracted from formalin-fixed paraffin-embedded material with the QIAamp DNA FFPE Tissue Kit (QIAGEN, Venlo, The Netherlands). Paired TURBT and cystectomy specimens underwent whole-exome sequencing (WES) and comprehensive genomic profiling with the TruSight Oncology 500 (TSO-500) panel, both on a NovaSeq 6000 instrument (Illumina, San Diego, CA, USA). WES reporting was restricted to a 64-gene bladder-cancer panel; TSO-500 data yielded single-nucleotide variants, small insertions, and deletions, focal amplifications, tumour mutational burden (TMB), and microsatellite-instability status. Variants of uncertain significance were recorded separately from pathogenic and likely pathogenic variants. Library preparation, sequencing parameters, bioinformatic pipelines, the gene list and all variant- and TMB-filtering thresholds are given in Supplementary Methods S1.

Tumour-cell content was estimated by the reporting pathologist on the corresponding haematoxylin-and-eosin slide for every paired case, specimens with low tumour cellularity have reduced sensitivity for variant detection.

### 2.5. Statistical Analysis

All statistical analyses were performed in Stata (StataNow/BE 18.5 for Mac, Apple Silicon; revision 26 February 2025; StataCorp LLC, College Station, TX, USA). Analyses were exploratory; no formal sample-size calculation was performed, because the cohort comprised all consecutive eligible patients treated in the study period.

Analysis populations were defined as follows. The response cohort comprises the 42 patients who underwent radical cystectomy and are therefore assessable for pathologic response; all comparisons between pCR and residual disease are restricted to this group. The 12 patients who did not undergo cystectomy are described separately and are not treated as a pathologic-response category, because they are a clinically heterogeneous group and their response status is unknown. The immune cohort comprises the 31 patients with an evaluable TURBT specimen, of whom 22 have paired cystectomy material. The genomic cohort comprises the 13 patients with adequate paired DNA, of whom 12 were evaluable for the alteration-level comparison, and 10 were fully evaluable at both timepoints. Patient numbers for every analysis are shown in Figure 1 and summarised in Supplementary Table S5.

Baseline characteristics were compared between patients achieving pCR vs. residual disease with Fisher’s exact test for binary variables, the Fisher–Freeman–Halton extension for larger contingency tables and the Mann–Whitney U test for continuous and ordinal variables. Proportions are reported with 95% Wilson score confidence intervals.

Paired changes in immune markers and TMB between TURBT and cystectomy were tested with Wilcoxon signed-rank tests, with the matched-pairs rank-biserial correlation as the effect size and the Hodges–Lehmann estimator as the location shift. Because the immune panel comprises eight markers tested simultaneously, *p*-values were additionally adjusted with the Benjamini–Hochberg false-discovery-rate procedure; both unadjusted *p*-values and adjusted q-values are reported, and inference is based on the adjusted values.

Agreement between the TSO-500 and WES estimates of TMB was quantified with the Spearman rank correlation, which was chosen in preference to the Pearson coefficient because TMB is right-skewed, the sample contains a single high-TMB observation with substantial leverage, and only eight patients had both assays at baseline. The Pearson coefficient is also reported for comparability with the published literature.

Overall survival (OS) was calculated from the start of NAC to death from any cause and recurrence-free survival (RFS) to the first of recurrence, progression or death; patients without an event were censored at last follow-up. Survival was estimated by the Kaplan–Meier method with 95% confidence intervals derived from Greenwood standard errors on the complementary log–log scale, and compared with the log-rank test. Survival comparisons were restricted to the operated cohort, in which pCR was compared with residual disease. Patients who did not undergo cystectomy are plotted for completeness but were not entered into any formal comparison, because they have no pathologic-response status. Associations with survival were further examined with Cox proportional-hazards models. Because only 11 deaths and 17 recurrence events were observed, multivariable models were restricted to three covariates (pathologic response, hydronephrosis, and clinical nodal status) to limit overfitting; even so, the number of events per variable remains below the conventional threshold of ten, and multivariable estimates are presented as exploratory, with the resulting imprecision made explicit. The proportional-hazards assumption was assessed on Schoenfeld residuals, and no violation was detected. A two-sided α of 0.05 was applied throughout.

## 3. Results

### 3.1. Patient Characteristics

Fifty-four consecutive patients were included; their flow through the study is shown in Figure 1. The cohort was predominantly male (43/54, 80%) with a median age at diagnosis of 67 years (IQR 56–71), and 44/54 (81%) completed the intended four cycles of dd-MVAC. Baseline characteristics according to pathologic response are shown in Table 1. Among the 42 operated patients, 11 (26%, 95% CI 15–41) achieved a pCR, and 31 had residual disease; 12 patients did not undergo cystectomy and are described separately because they are not assessable for pathologic response. Hydronephrosis at diagnosis was the only baseline variable associated with the absence of pCR (1/11, 9% in the pCR group vs. 16/31, 52% in the residual-disease group; *p* = 0.016). Age, sex, smoking status, clinical T and N stage, previous intravesical BCG, completion of four chemotherapy cycles, and variant histology did not differ significantly between response groups. Time from TURBT to the start of chemotherapy was nominally longer in patients who achieved a pCR (median 35 vs. 29 days; *p* = 0.026), but this difference does not survive correction for the eleven comparisons made in Table 1 (q = 0.29), and we regard it as hypothesis-generating rather than a genuine determinant of response.

Forty-four patients (81%) completed all four planned cycles; nine received fewer (three cycles, n = 3; two cycles, n = 4; one cycle, n = 2), and one received six. Treatment was curtailed for haematological or renal toxicity, deteriorating performance status or radiological progression, and 17 patients (31%) required at least one hospital admission for chemotherapy-related toxicity. Twelve patients did not proceed to cystectomy: four had progressive disease during chemotherapy, four were managed with TURBT alone because of patient preference or unfitness for surgery at re-assessment, two received primary chemoradiotherapy, and one received primary radiotherapy as a bladder-preserving alternative, and one was re-classified as upper-tract urothelial carcinoma. All 54 tumours were urothelial carcinoma; variant differentiation was present in five (squamous, n = 4; micropapillary, n = 1). Twelve patients had previously been treated for non-muscle-invasive disease, and eight had received intravesical BCG.

### 3.2. Patient Trajectories and Survival

**Figure 2:**
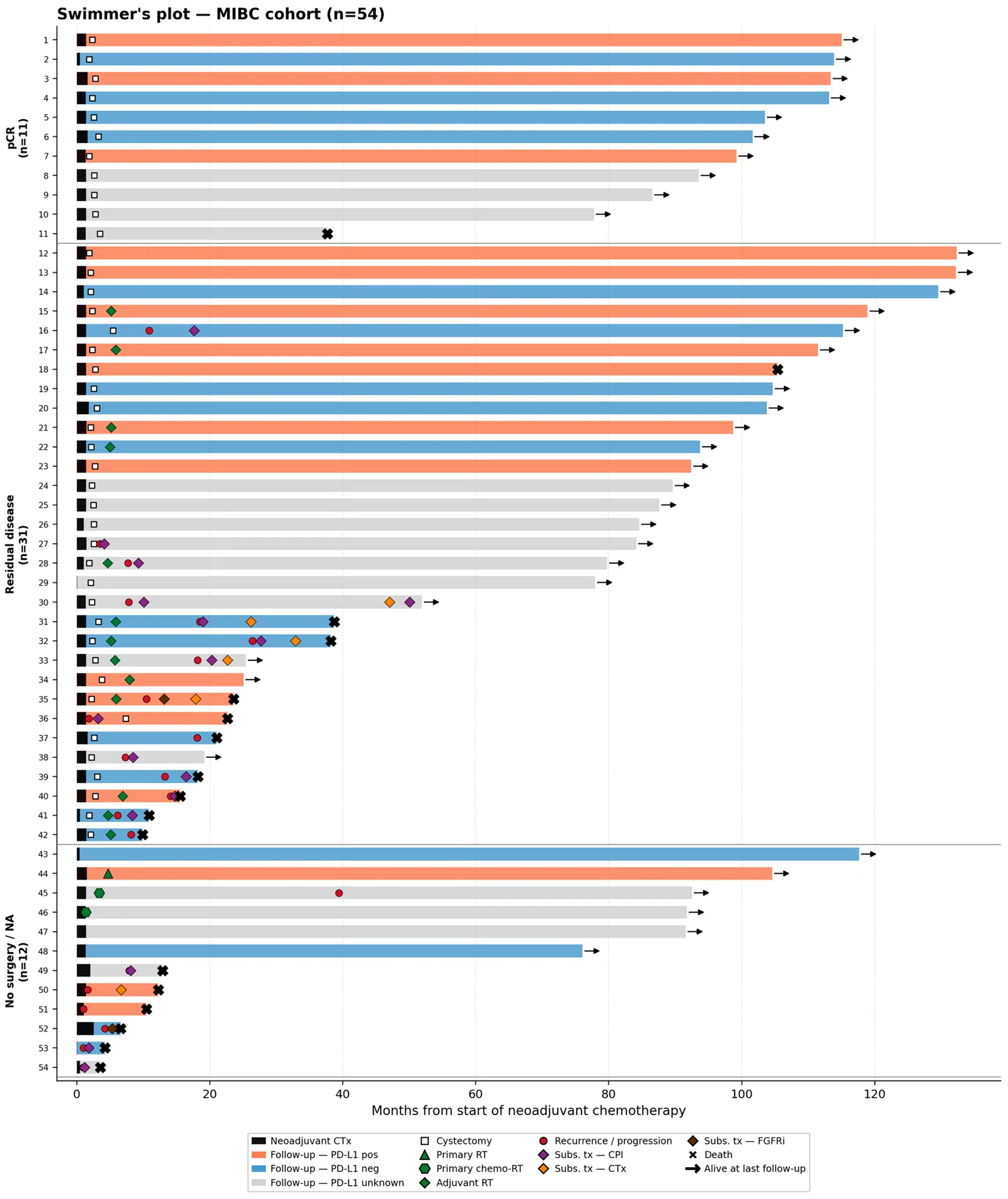
Swimmer’s plot of the 54-patient MIBC cohort, grouped by pathologic response (pCR, residual disease, no surgery) and coloured by PD-L1 status. Black segments indicate neoadjuvant dd-MVAC; markers denote cystectomy, adjuvant radiotherapy (operated patients), primary radiotherapy or chemo-radiotherapy (no-surgery group), recurrence, subsequent systemic therapy lines, death, and alive-at-last-follow-up.

Individual patient trajectories are shown in the swimmer’s plot (Figure 2). Median follow-up from the start of NAC was 87 months (IQR 24–104) for the whole cohort and 94 months among survivors. Twenty-two patients (41%) experienced disease recurrence or progression and 17 (31%) died. Outcomes diverged sharply by pathologic response: no recurrences and a single death occurred among the 11 patients with a pCR (median follow-up 102 months), compared with 15 recurrences and 10 deaths among the 31 patients with residual disease (median follow-up 84 months). The 12 patients who did not undergo cystectomy had the poorest outcomes (7 recurrences, 6 deaths; median follow-up 45 months). Twenty patients received a second line of therapy, six a third line, and three a fourth line, most commonly checkpoint inhibitors followed by platinum-based or taxane chemotherapy. Of the 12 patients who did not undergo cystectomy, three received primary radiotherapy (n = 1) or primary chemo-radiotherapy (n = 2) as their definitive local treatment.

**Figure 3:**
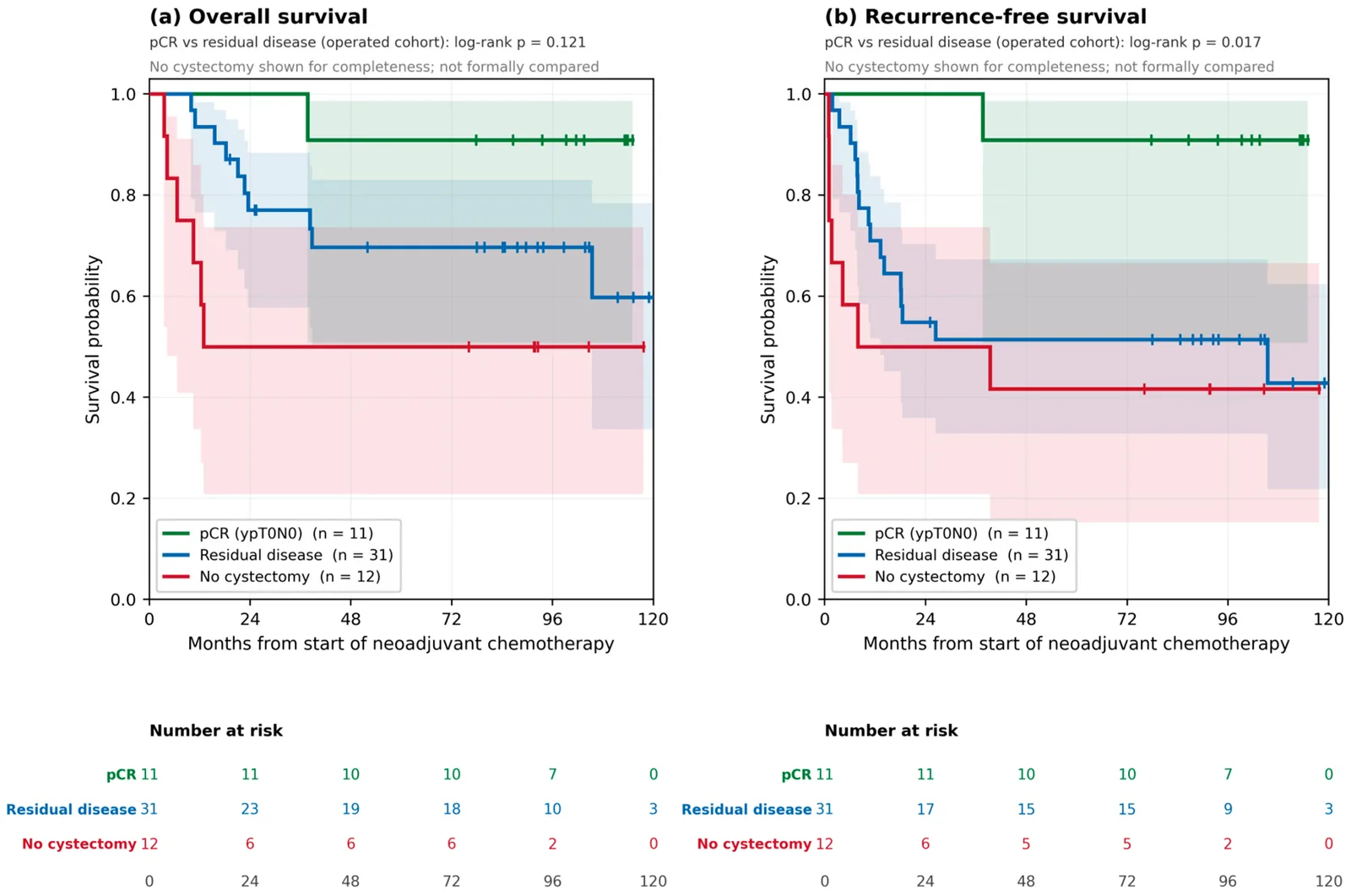
Kaplan–Meier estimates of (**a**) overall survival and (**b**) recurrence-free survival from the start of neoadjuvant chemotherapy. Shaded bands are 95% confidence intervals (Greenwood standard error, log–log transformed); tick marks indicate censoring; numbers at risk are shown beneath each panel. The comparison shown is between the two operated groups (pCR vs. residual disease). The no-cystectomy group is plotted for completeness but was not formally compared, because these patients have no pathologic-response status.

Within the operated cohort, patients achieving a pCR had significantly longer recurrence-free survival than those with residual disease (log-rank *p* = 0.017), whereas the difference in overall survival did not reach statistical significance (*p* = 0.121; Figure 3). Patients who did not undergo cystectomy are shown on the same axes for completeness but were not compared formally, since their pathologic-response status is unknown; their poor outcome reflects a mixture of progression on chemotherapy, unfitness for surgery, and bladder preservation rather than chemoresistance alone. Five-year overall survival was 91% (95% CI 51–99) after pCR, 70% (95% CI 50–83) with residual disease, and 50% (95% CI 21–74) in non-operated patients; the corresponding five-year recurrence-free survival was 91% (95% CI 51–99), 51% (95% CI 33–67), and 42% (95% CI 15–67). The width of these intervals is a direct consequence of the small number of events per group.

In univariable Cox models restricted to operated patients, residual disease was associated with recurrence (HR 8.02, 95% CI 1.06–60.68; *p* = 0.044) but not significantly with death (HR 4.43, 95% CI 0.57–34.70; *p* = 0.156). In multivariable models adjusted for hydronephrosis and clinical nodal status, the point estimates were essentially unchanged, but neither association reached significance (RFS HR 6.97, 95% CI 0.88–54.99, *p* = 0.065; OS HR 5.22, 95% CI 0.64–42.69, *p* = 0.123; Table 2). With 11 deaths and 17 recurrence events, these models are underpowered—fewer than six events per variable—and the very wide confidence intervals should be read as a statement about the precision of this cohort rather than as evidence that pathologic response is unimportant.

Adjuvant radiotherapy was administered to 13 of the 31 patients with residual disease and to none of the patients achieving a pCR, reflecting its use as a response-directed rather than a baseline treatment decision; two further patients in the no-cystectomy group received radiotherapy as their primary local treatment.

### 3.3. Immune-Related Characteristics and Effect of Neoadjuvant Chemotherapy

Baseline (TURBT) immune characteristics were not associated with pathologic response. Median CD3, CD8, FOXP3, PD-1, PD-L2, and NY-ESO-1 grades were comparable between the 7 evaluable patients who achieved a pCR and the 18 who did not (all unadjusted *p* ≥ 0.44; all q = 0.92, Mann–Whitney U with Benjamini–Hochberg correction; Table S4). PD-L1 positivity, assessed with the combined variable, was likewise not associated with pCR (3/7 evaluable pCR patients vs. 11/22 without pCR; *p* > 0.99). With only seven evaluable responders, this analysis has very low power: a difference of the size usually considered clinically meaningful would often have been missed, so these are non-informative rather than negative findings.

In contrast, neoadjuvant dd-MVAC measurably remodelled the tumour immune microenvironment (Table 3). In paired TURBT–cystectomy comparisons, intratumoral FOXP3 fell in 16 of 22 paired cases with no case increasing (*p* < 0.001, q = 0.001), NY-ESO-1 in 10 of 21 with none increasing (*p* = 0.004, q = 0.013), PD-L2 in 10 of 22 (*p* = 0.007, q = 0.013), and CD3 in 10 of 22 (*p* = 0.029, q = 0.043). All four remain significant after Benjamini–Hochberg correction across the six markers examined. CD8 did not change significantly (*p* = 0.351) and PD-1, which was negative or near-negative in most specimens at both timepoints, showed no significant change (*p* = 0.174). PD-L1 status was relatively stable: of 22 paired cases, 4 remained positive, and 4 lost positivity, while 2 of 14 baseline-negative tumours gained PD-L1 expression after NAC. Because these paired specimens come almost exclusively from patients with residual disease, these changes describe the immune consequences of chemotherapy in chemoresistant tumours and cannot be generalised to responding tumours, for which no post-treatment tissue exists.

### 3.4. Effect of Neoadjuvant Chemotherapy on Tumour Mutational Burden

**Figure 4:**
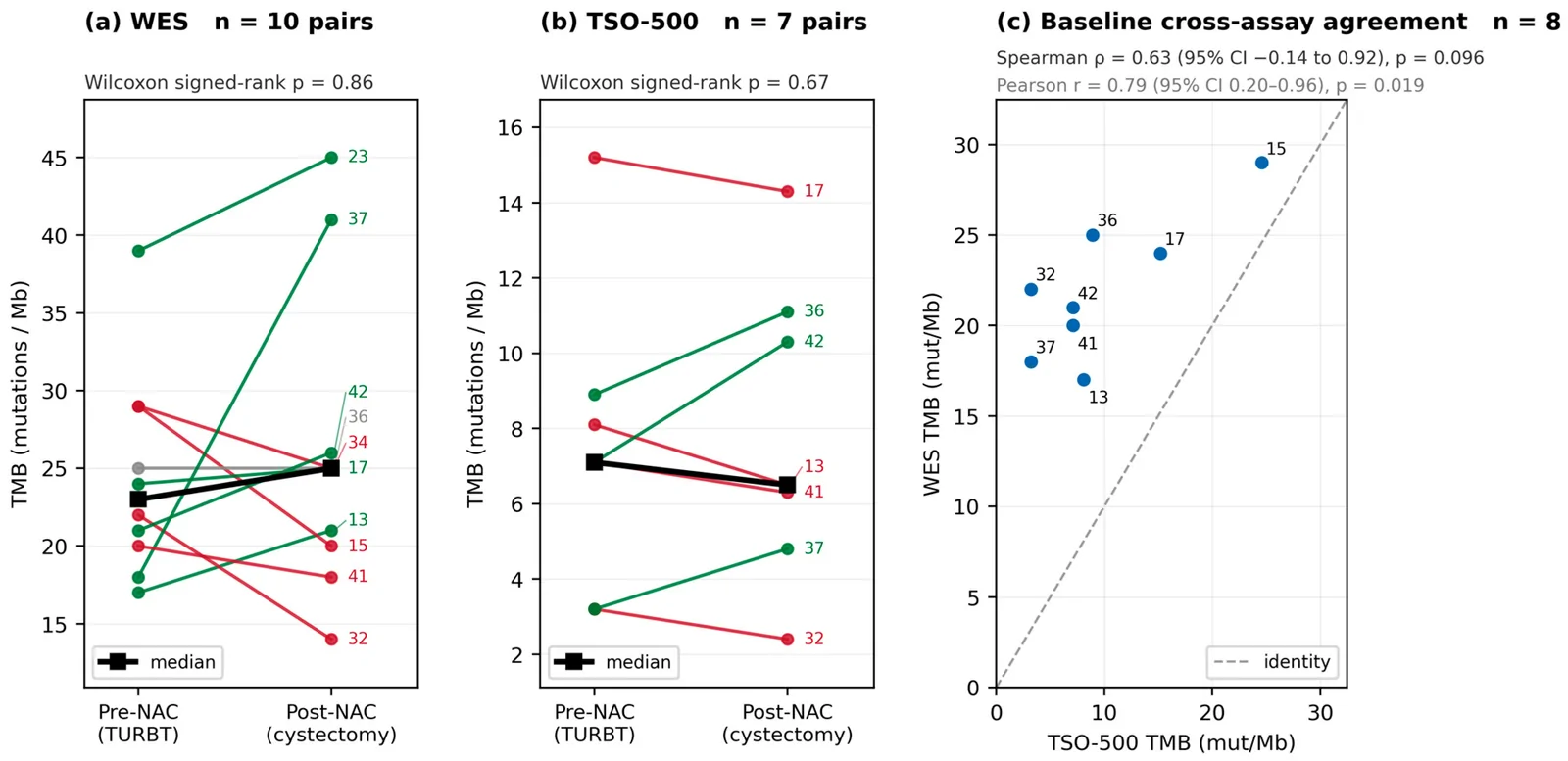
Tumour mutational burden (TMB) before and after neoadjuvant chemotherapy. (**a**) Paired whole-exome sequencing (WES), n = 10 pairs. (**b**) Paired TSO-500 comprehensive genomic profiling, n = 7 pairs. Each line is one patient, coloured green for an increase and red for a decrease; the heavy black line is the median. Patients are identified by the same number as in Figure 2. (**c**) Agreement between the two assays at baseline, n = 8 patients with both assays; the dashed line is the line of identity. Because TMB is skewed and the sample is small, the Spearman rank correlation is the primary estimate, with the Pearson coefficient shown for comparison.

TMB was assessed in paired samples by CGP using TSO-500 and by WES. TMB did not change significantly after neoadjuvant chemotherapy by either assay (TSO-500: median 7.1 pre-NAC vs. 6.5 post-NAC, n = 7 pairs, Wilcoxon *p* = 0.67; WES: median 23 vs. 25, n = 10 pairs, *p* = 0.86; Figure 4). Changes were heterogeneous and bidirectional, with no consistent direction of effect at the cohort level. Agreement between the two assays at baseline was moderate and not statistically significant in the eight patients assayed by both methods (Spearman ρ = 0.63, 95% CI −0.14 to 0.92, *p* = 0.096; Pearson r = 0.79, 95% CI 0.20–0.96, *p* = 0.019), and WES returned systematically higher absolute values than TSO-500, so the two assays should not be used interchangeably. With seven and ten pairs, respectively, these analyses can exclude only very large systematic shifts in TMB; they do not establish that TMB is unchanged by chemotherapy. 

### 3.5. Effect of Neoadjuvant Chemotherapy on Genetic Alterations

**Figure 5:**
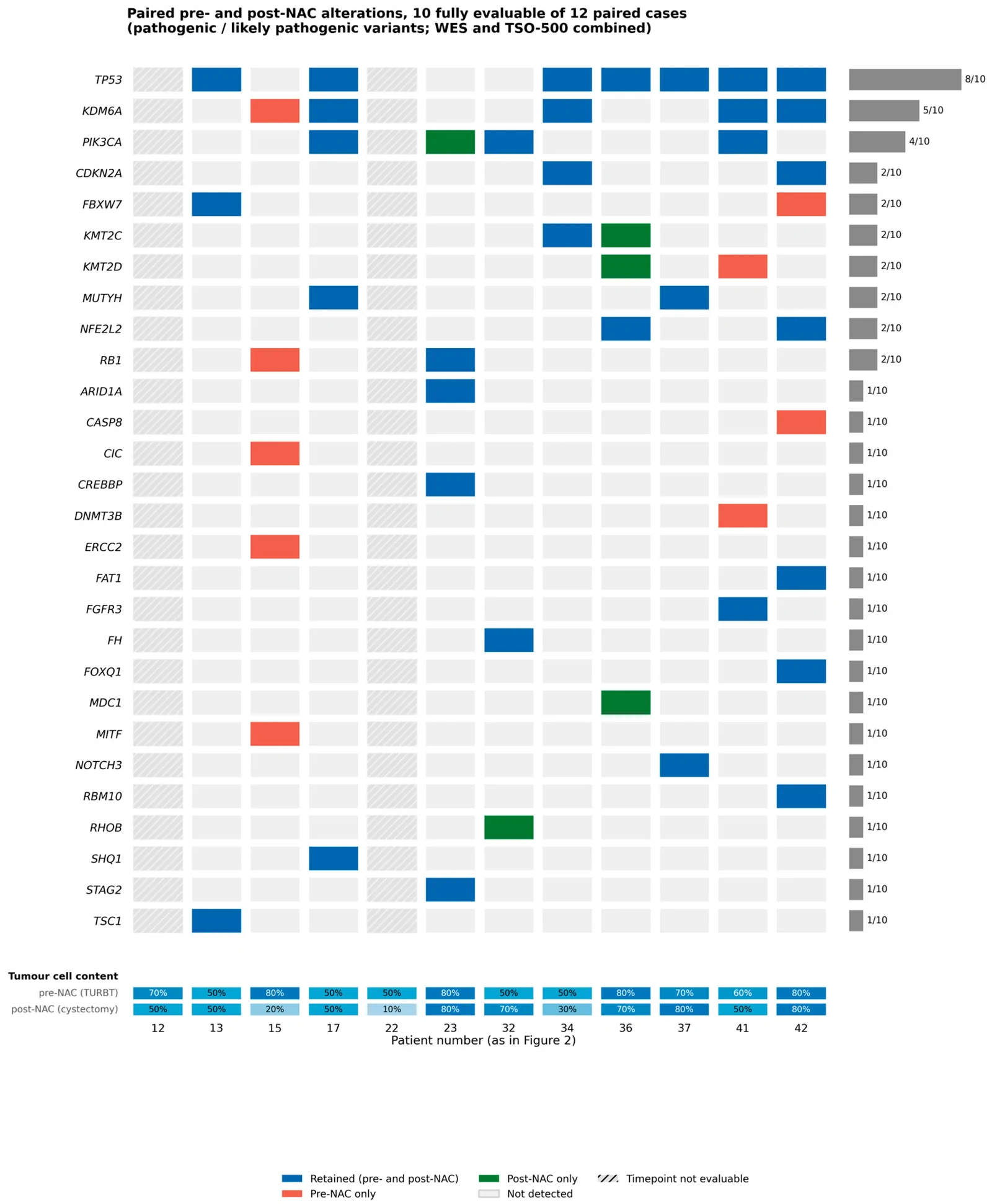
Oncoprint of paired pre- and post-NAC alterations. Each column is one patient, numbered as in Figure 2, and each row one gene; only pathogenic or likely pathogenic variants are shown (variants of uncertain significance are excluded), combining WES and TSO-500 calls. Ten of the twelve paired cases were fully evaluable at both timepoints; the two cases in which one timepoint failed sequencing are hatched. The annotation track shows tumour-cell content of each specimen, which is systematically lower in post-NAC material and is the most likely explanation for apparent variant loss. The full variant-level listing is provided in Supplementary Table S2. Amplification copy numbers shown in parentheses. “/” indicates not evaluable; “None detected” indicates no amplification identified. TSO-500, TruSight Oncology 500 panel; WES, whole-exome sequencing; NAC, neoadjuvant chemotherapy.

Paired pre- and post-NAC mutational profiles were available for 12 cases, of which 10 were fully evaluable at both timepoints (Figure 5); in one case, the post-NAC specimen and in another both specimens failed sequencing. *TP53* was the most frequently altered gene (8 of 10 fully evaluable cases), followed by *KDM6A* (5 of 10) and *PIK3CA* (4 of 10), with further alterations in the chromatin-remodelling genes *KMT2C* and *KMT2D*. An activating *FGFR3* alteration was identified in one case and an *ERCC2* mutation—associated with cisplatin sensitivity—in another. Focal amplifications (*MYC*, *CCNE1*, *EGFR*, *MET*, *CCND1*) were detected by TSO-500 in three cases, with amplification copy number generally decreasing in the post-NAC sample. The dominant clonal driver mutations were retained after chemotherapy in every fully evaluable case, and most discordances involved variants of uncertain significance or changes in variant allele frequency rather than truncal drivers, consistent with the TCGA molecular characterisation of MIBC [20]. Apparent variant loss was concentrated in the specimens with the lowest post-NAC tumour-cell content (as low as 10–20%; annotation track in Figure 5), so reduced tumour cellularity after treatment—rather than true clonal elimination—is the most parsimonious explanation for these differences.

## 4. Discussion

This cohort of 54 patients with MIBC treated with neoadjuvant dd-MVAC, followed for a median of more than seven years, offers a view of what platinum does and does not achieve. Long-term outcome was strongly stratified by pathologic response, although overall survival within the operated cohort did not differ significantly. Chemotherapy depleted intratumoral FOXP3, CD3, PD-L2, and NY-ESO-1 populations, while tumour mutational burden and driver alterations remained broadly concordant. No pre-treatment immune or genomic marker distinguished platinum-refractory from platinum-sensitive tumours.

### 4.1. Long-Term Outcomes by Pathologic Response

Patients achieving a pCR had a benign course, with no recurrences and a single death over a median follow-up exceeding eight years. Their recurrence-free survival advantage over patients with residual disease was significant (*p* = 0.017), whereas the overall-survival difference was not (*p* = 0.121), and the multivariable hazard ratios carry confidence intervals spanning an order of magnitude. We report the operated-only comparison as primary because patients who did not undergo cystectomy have no pathologic-response status; they comprise progressors, patients unfit for surgery and patients who chose bladder preservation, so attributing their poor outcome to chemoresistance would conflate treatment selection with tumour biology. In an exploratory analysis, eight further patients downstaged to non-muscle-invasive disease (ypT1/Tis/Ta) had overall survival indistinguishable from the pCR group (*p* = 0.91), consistent with the literature treating post-NAC ≤ ypT1 ypN0 as a near-complete response and broadening the apparently platinum-sensitive subset from 26% to 45% of operated patients.

### 4.2. Chemotherapy-Induced Immune Remodelling

Cisplatin remains a very active agent in urothelial cancer, forming platinum–DNA adducts that block transcription and induce apoptosis; resistance arises chiefly from enhanced DNA-damage repair, notably nucleotide-excision repair involving ERCC2 [21], from increased glutathione conjugation and from reduced drug uptake [22,23,24]. Carboplatin is substantially less potent and performs worse in combination [25], so the value of cisplatin rests on combination regimens, among which dd-MVAC outperformed gemcitabine–cisplatin in the VESPER trial with a demonstrable dose–response relationship [6,26,27,28]. Combining platinum with checkpoint inhibition is mechanistically attractive, since platinum induces immunogenic cell death, releases tumour antigens and can convert “cold” tumours into inflamed ones [29,30,31], and this rationale has produced consistent perioperative benefit across several tumour types [32,33,34,35,36,37]. In urothelial cancer, however, only NIAGARA [8] has been positive, whereas ENERGIZE and KEYNOTE-866 have not, indicating that the interaction is context-dependent rather than simply additive.

**Figure 6:**
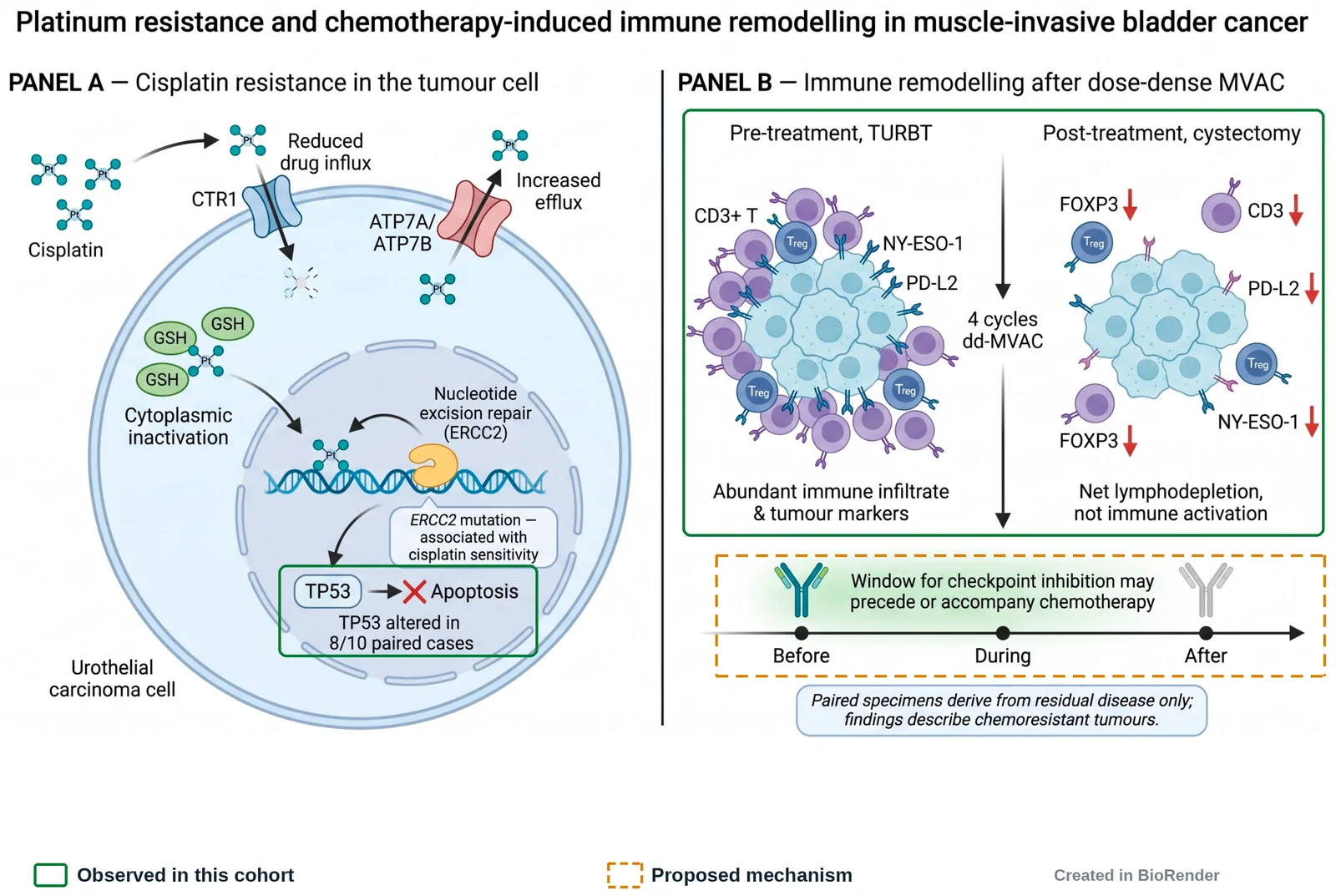
Mechanisms of platinum resistance and chemotherapy-induced immune remodelling in muscle-invasive bladder cancer. (**A**) panel: determinants of cisplatin sensitivity within the tumour cell: reduced drug influx through CTR1, increased efflux via ATP7A and ATP7B, cytoplasmic inactivation by glutathione conjugation, and repair of platinum–DNA adducts by the nucleotide-excision repair pathway, in which ERCC2 alteration has been associated with cisplatin sensitivity; TP53 alteration, present in 8 of 10 fully evaluable paired cases in this cohort, permits evasion of apoptosis. (**B**) panel: immune remodelling observed after four cycles of dd-MVAC, with reductions in FOXP3, CD3, PD-L2, and NY-ESO-1 indicating net lymphodepletion rather than immune activation, and the resulting implication that the window for checkpoint inhibition may precede or accompany chemotherapy rather than follow it. Paired specimens derive exclusively from patients with residual disease, so the right panel characterises chemoresistant tumours and cannot be generalised to responders.

Against that background (Figure 6), the lymphocytic attrition we observed after dd-MVAC—affecting regulatory (FOXP3) and total (CD3) T-cell populations as well as PD-L2 and NY-ESO-1—is notable. It supports the regulatory-T-cell-depletion element of that account, since FOXP3^+^ cells fell in every discordant pair, but not a net immunostimulatory effect, since the total CD3^+^ infiltrate fell in parallel. Dose-dense MVAC is markedly myelosuppressive, and sustained lymphodepletion over four cycles plausibly removes effector and regulatory lymphocytes alike, leaving a less inflamed microenvironment at surgery; the concomitant loss of the cancer–testis antigen NY-ESO-1, a substrate for antigen-specific T-cell responses, points the same way. If correct, this would place the window for checkpoint inhibition during or before chemotherapy rather than after it, and may contribute to the divergent trial results noted above. The interpretation remains speculative: the observations are associative, derive from 22 paired specimens, and lack functional or transcriptomic corroboration.

### 4.3. Genomic Concordance and the Limits of Paired Residual-Tumour Analysis

The stability of TMB and of driver alterations accords with the view that platinum resistance in urothelial cancer is not predominantly acquired through genomic evolution over a short neoadjuvant course. The determinants of cisplatin sensitivity outlined above are likely present from the outset, which supports pre-treatment profiling as the appropriate window for biomarker-driven selection. Molecular subtype has been associated with differential benefit from NAC [20,38], and combining subtype with DNA-damage-response status may outperform any single marker.

Two constraints bound this conclusion. First, the paired analyses carry a structural selection bias: post-treatment tissue exists only where tumour remained, so patients achieving a pCR are excluded by definition, and most early progressors never reached cystectomy. Observed concordance therefore describes the stability of resistant clones; it cannot show that chemotherapy fails to eliminate sensitive subclones, because in responding tumours that elimination leaves nothing to sequence. Second, post-NAC specimens had systematically lower tumour-cell content, in some cases only 10–20%, which reduces variant-detection sensitivity and can mimic clonal loss; the apparent variant losses cluster in precisely these low-purity specimens and are best read as analytical rather than biological. With 10 paired cases for the alteration comparison and 7 to 10 for TMB, we can exclude only very large systematic shifts, not subclonal evolution of the kind demonstrated by deeper sequencing after chemotherapy in urothelial and other carcinomas. These data are compatible with, but do not establish, genomic stability under platinum.

### 4.4. Clinical Implications

The absence of an association between baseline PD-L1, or any other immune marker, and pCR echoes ABACUS and PURE-01, in which PD-L1 enriched for but did not reliably predict response [39,40]. With seven evaluable responders, our analysis had power only for very large effects, so no association was detected rather than none exists. Prospective validation will require pre-specified biomarker endpoints, pre-treatment tissue from all patients rather than only those with residual disease, and circulating tumour DNA endpoints, since a pathologic complete response by definition leaves no tissue to analyse.

Any bearing on treatment sequencing is indirect and should be distinguished from what we measured: this cohort was treated between 2013 and 2019, and no patient received an antibody–drug conjugate or perioperative checkpoint inhibitor. Patients achieving a pCR on dd-MVAC have an excellent long-term outcome, which argues against abandoning platinum for all patients as EV–pembrolizumab moves into the perioperative setting [7], and which is directly relevant where EV–pembrolizumab is unavailable or where patients cannot receive it. Since no pre-treatment marker has identified responders, a pragmatic approach is to use pathologic response itself as the biomarker: proceed with platinum in cisplatin-eligible patients and reserve intensified or alternative therapy for those with residual disease at cystectomy, in whom adjuvant checkpoint inhibition already has trial support [8,9,10,11]. In locally advanced and metastatic disease, platinum retains a defined role in later lines after first-line ADC-based regimens [12,13,14], so persistent platinum sensitivity remains clinically relevant across the treatment trajectory.

### 4.5. Limitations

This study is retrospective, single-centre and modest in size: 54 patients, 42 assessable for pathologic response and fewer in each translational analysis. Survival comparisons rest on 11 deaths and 17 recurrence events, giving fewer than six events per variable even in the restricted multivariable models, so estimates are imprecise, and the probability of a type II error is high; a non-significant result here is not evidence of no effect. The paired immune and genomic subsets are small and non-randomly selected towards chemoresistant disease. Immune infiltration was assessed semi-quantitatively by two observers rather than by digital pathology, and because only consensus scores were retained, a formal inter-observer agreement statistic could not be computed. Marker comparisons were corrected for multiplicity, but the study remains exploratory, and no biomarker finding should be regarded as validated. Against these constraints, the strengths are a uniformly treated consecutive cohort, long and near-complete follow-up, and paired pre- and post-treatment tissue.

## 5. Conclusions

In this long-term cohort, neoadjuvant dd-MVAC was associated with measurable depletion of intratumoral regulatory and total T-cell populations and of PD-L2 and NY-ESO-1 expression, while paired tumour mutational burden and driver alterations remained broadly concordant. Because post-treatment tissue exists only for patients with residual disease, these observations characterise chemoresistant tumours and cannot be generalised to responders. No pre-treatment immune or genomic marker, including PD-L1, identified platinum-refractory patients, but the evaluable subsets were small enough that this represents absence of evidence rather than evidence of absence. Patients achieving a pathologic complete response experienced almost no recurrences over more than seven years, a reminder that platinum still serves a subgroup of patients well as antibody–drug conjugate and immunotherapy regimens reshape perioperative care. Identifying that subgroup before treatment will require prospective studies with pre-specified biomarker endpoints, universal pre-treatment tissue collection and circulating tumour DNA.

## Figures and Tables

**Table 1:** Baseline characteristics by pathologic response.

Characteristic	pCR (n = 11)	Residual Disease (n = 31)	No Cystectomy (n = 12)	*p*-Value *
Age at TURBT, years—median (IQR)	68.9 (56.5–70.6)	64.2 (56.4–72.3)	66.8 (50.2–69.9)	0.864
Male sex	9/11 (82%)	23/31 (74%)	11/12 (92%)	>0.99
Smoking—never/former/active	3/4/4	10/11/10	3/6/2	>0.99
Clinical stage cT3–4 (vs. cT2)	7/11 (64%)	24/31 (77%)	8/12 (67%)	0.437
Clinical node positive (cN+)	3/11 (27%)	12/31 (39%)	8/12 (67%)	0.717
Hydronephrosis at diagnosis	1/11 (9%)	16/31 (52%)	4/12 (33%)	0.016
Previous intravesical BCG	1/11 (9%)	4/31 (13%)	3/12 (25%)	>0.99
Variant histology (any)	1/11 (9%)	4/31 (13%)	0/12 (0%)	>0.99
Completed 4 dd-MVAC cycles	10/11 (91%)	27/31 (87%)	7/12 (58%)	>0.99
Days from TURBT to start of NAC—median (IQR)	35 (32–60)	29 (20–38)	26 (20–41)	0.026
Days from end of NAC to cystectomy—median (IQR)	37 (32–43)	33 (26–43)	—	0.439

* *p*-values compare the two operated groups only (pCR vs. residual disease); the no-cystectomy column is descriptive and was not included in any significance test, because these patients are not assessable for pathologic response and form a clinically heterogeneous group. Fisher’s exact test was used for binary variables, the Fisher–Freeman–Halton extension for the 3 × 2 smoking table and the Mann–Whitney U test for continuous variables; *p*-values that round to 1.000 are shown as >0.99. Exact proportions with 95% Wilson confidence intervals are given in Table S3. With eleven comparisons in this table, the difference in time from TURBT to NAC (*p* = 0.026) does not survive Benjamini–Hochberg correction (q = 0.29) and is reported as hypothesis-generating only.

**Table 2:** Univariable and multivariable Cox proportional-hazards models for overall and recurrence-free survival in operated patients.

Variable	OS—HR (95% CI)	*p*	RFS—HR (95% CI)	*p*
Univariable				
Residual disease (vs. pCR)	4.43 (0.57–34.70)	0.156	8.02 (1.06–60.68)	0.044
Hydronephrosis	0.93 (0.27–3.20)	0.914	2.15 (0.82–5.59)	0.118
Clinical stage cT3–4	1.39 (0.30–6.43)	0.677	1.19 (0.39–3.66)	0.760
Clinical node positive	1.44 (0.44–4.72)	0.546	1.71 (0.66–4.42)	0.272
Completed 4 dd-MVAC cycles	1.29 (0.16–10.08)	0.809	0.85 (0.19–3.70)	0.824
Age (per year)	1.02 (0.95–1.09)	0.566	0.99 (0.94–1.04)	0.738
Multivariable †				
Residual disease (vs. pCR)	5.22 (0.64–42.69)	0.123	6.97 (0.88–54.99)	0.065
Hydronephrosis	0.51 (0.12–2.12)	0.357	1.28 (0.44–3.79)	0.650
Clinical node positive	1.63 (0.42–6.27)	0.476	1.31 (0.45–3.78)	0.621

Cox proportional-hazards models restricted to the 42 operated patients. OS, overall survival (11 events); RFS, recurrence-free survival (17 events); HR, hazard ratio. † The multivariable model was deliberately limited to three covariates. The confidence intervals are correspondingly wide, and these estimates should be regarded as exploratory. The model was not significant for OS (likelihood-ratio *p* = 0.267) and was significant for RFS (*p* = 0.040). The number of patients and events in each covariate category is given in Supplementary Table S8.

**Table 3:** Paired changes in immune markers after neoadjuvant dd-MVAC chemotherapy.

Marker	Baseline n	Paired n	Pre-NAC Median (IQR)	Post-NAC Median (IQR)	Effect Size r	*p*	q *
FOXP3	31	22	Low (Low–Low)	None (None–Low)	−1.00	<0.001	0.001
NY-ESO-1	30	21	Low (None–Low)	None (None–None)	−1.00	0.004	0.013
PD-L2 (% cells)	31	22	0 (0–4.5)	0 (0–0)	−0.94	0.007	0.013
CD3	31	22	Moderate (Mod–Mod)	Moderate (Low–Mod)	−0.67	0.029	0.043
PD-1 (% cells)	31	22	0 (0–0)	0 (0–0)	−1.00	0.174	0.208
CD8	31	21	Low (Low–Mod)	Low (Low–Mod)	−0.33	0.351	0.351

Paired TURBT–cystectomy comparisons, Wilcoxon signed-rank test. CD3, CD8, FOXP3, and NY-ESO-1 were assessed as a four-level semi-quantitative grade (none, low, moderate, strong), coded 0–3 for analysis; PD-1 and PD-L2 as the percentage of positive cells. Mod, moderate. The effect size r is the matched-pairs rank-biserial correlation, where −1 indicates that every discordant pair moved downwards. * q = Benjamini–Hochberg false-discovery-rate-adjusted *p*-value across the six markers. FOXP3, NY-ESO-1, PD-L2, and CD3 all remain significant after correction. Baseline grades were not associated with pathologic response (all q = 0.92; Table S4).

## Data Availability

The data presented in this study are available on request from the corresponding author. The data are not publicly available owing to privacy and ethical restrictions.

## Supplementary Materials

The following supporting information can be downloaded at: https://www.mdpi.com/article/10.3390/cancers18172839/s1, Supplementary Methods S1—Detailed wet-lab and bioinformatic methods for genomic profiling; Supplementary Methods S2—Immunohistochemistry scoring detailed criteria; Table S1—Antibodies and staining conditions used for immunohistochemistry; Table S2—Paired pre- and post-NAC genetic alterations, variant-level detail; Table S3—Baseline proportions with 95% confidence intervals; Table S4—Baseline immune markers by pathologic response; Table S5—Analysis populations and missing data; Table S6—Sensitivity analysis using the averaged field score; Table S7—Molecular data availability per paired case; Table S8—Patients and events per covariate category.

## Abbreviations

The following abbreviations are used in this manuscript:

**Abbreviation**	**Definition**
ADC	antibody–drug conjugate
BCG	bacillus Calmette–Guérin
CGP	comprehensive genomic profiling
CI	confidence interval
CPS	combined positive score
DAB	diaminobenzidine
dd-MVAC	dose-dense methotrexate, vinblastine, doxorubicin and cisplatin
ECOG	Eastern Cooperative Oncology Group
EV	enfortumab vedotin
FDR	false discovery rate
FFPE	formalin-fixed paraffin-embedded
HR	hazard ratio
ICI	immune checkpoint inhibitor
IHC	immunohistochemistry
IQR	interquartile range
ITE	impossible to evaluate
la/mUC	locally advanced or metastatic urothelial carcinoma
MIBC	muscle-invasive bladder cancer
MSI	microsatellite instability
NAC	neoadjuvant chemotherapy
OS	overall survival
PBC	platinum-based chemotherapy
pCR	pathologic complete response
RFS	recurrence-free survival
RT	radiotherapy
TC	tumour cell (score)
TIL	tumour-infiltrating lymphocyte
TMB	tumour mutational burden
TSO-500	TruSight Oncology 500
TURBT	transurethral resection of bladder tumour
UC	urothelial carcinoma
UTUC	upper-tract urothelial carcinoma
VAF	variant allele frequency
VUS	variant of uncertain significance
WES	whole-exome sequencing
