# Supplementary material for: Long-Term Outcomes and Paired Immune and Genomic Exploratory Analyses After Neoadjuvant Dose-Dense MVAC in Muscle-Invasive Bladder Cancer: A Single-Centre Case Series
Source: Cancers (Basel). 2026 Sep 2;18(17):2839. doi: 10.3390/cancers18172839 (PMC13565745; doi:10.3390/cancers18172839)
Supplement: Supplementary file 1 [file cancers-18-02839-s001.zip › cancers-4424674-supplementary.pdf]

Supplementary Materials

Supplementary Methods S1. Detailed wet-lab and bioinformatic methods for genomic profiling

S1.1. DNA and RNA extraction

Macro-dissection of representative tumour areas (≥30% tumour cellularity, marked by a uropathologist on a hematoxylin-and-eosin slide) was performed on 4–10-µm sections of the formalin-fixed, paraffin-embedded TURBT and cystectomy blocks. Genomic DNA was extracted using the *QIAamp DNA FFPE Tissue Kit* (Qiagen, Hilden, Germany) and RNA using the *RNeasy FFPE Kit* (Qiagen) according to the manufacturer's instructions. Matched germline DNA was extracted from non-neoplastic tissue (or from peripheral blood mononuclear cells where available) using the same kit. DNA and RNA yield were quantified with a Qubit fluorometer (Thermo Fisher Scientific, Waltham, MA, USA) and DNA integrity assessed by Δ Cq on the Illumina TruSight Oncology 500 Library Quality Control assay (FFPE QC).

S1.2. Comprehensive Genomic Profiling (TSO-500)

Comprehensive genomic profiling was performed with the Illumina TruSight Oncology 500 (TSO-500) hybrid-capture assay (Illumina Inc., San Diego, CA, USA), a 1.94-Mb panel targeting single-nucleotide variants and small insertions/deletions in 523 cancer-associated genes, copy-number variants in 59 genes, fusions involving 55 driver genes, and on-panel tumour mutational burden (TMB) and microsatellite-instability (MSI) measurement. Libraries were prepared from 40–80 ng of FFPE-derived DNA and 40–80 ng of FFPE-derived RNA using the TSO-500 sample-preparation protocol, as previously validated for diagnostic implementation within the Belgian PRECISION/BALLETT consortium [Froyen et al., *Cancers* 2022; Maes et al., *npj Precis. Oncol.* 2025]. Pooled libraries (8 DNA + 8 RNA samples per run) were sequenced on an Illumina *[NextSeq 550Dx]* instrument with a High-Output v2.5 (300 cycles) flow cell at 2 × 101 bp paired-end. Reported analytical performance of this implementation includes sensitivity and specificity ≥ 99% for SNVs, indels, CNVs, fusions and MSI; a limit of detection well below 5% variant allele frequency; and divergent clinical interpretation only for TMB values close to the 10 mut/Mb threshold.

Raw sequencing data were analysed with the Illumina TSO-500 Local App software version 2.2; samples processed from 2023 onward were analysed on an on-premise DRAGEN server with the DRAGEN TSO-500 Analysis Software version 2.1. TMB was calculated as the number of non-synonymous somatic variants (missense, nonsense, frameshift, splice-site) per coding megabase, after exclusion of variants annotated as common in gnomAD or as germline by the

panel's tumour-only filtering pipeline. MSI was scored using the panel-native MSI algorithm; positive cases were corroborated against WES-derived MSIsensor-pro calls (S1.3).

S1.3. Whole-Exome Sequencing (WES)

In parallel, paired tumour and germline DNA from the 13 evaluable cases was subjected to whole-exome sequencing using the *(Twist Comprehensive Exome Panel — Twist Bioscience, San Francisco, CA)* hybrid-capture kit, with input of 100–200 ng genomic DNA. Indexed libraries were sequenced on an Illumina NovaSeq 6000 platform at 2 × 150 bp paired-end to a mean on-target coverage of approximately *200*× for tumour samples and *100*× for matched germline samples.

Reads were aligned to GRCh38 with BWA-MEM (Burrows–Wheeler Aligner v0.7.17); duplicates marked with Picard MarkDuplicates (Broad Institute); base-quality recalibration performed with GATK BaseRecalibrator. Somatic single-nucleotide variants and small insertions/deletions were called with GATK4 Mutect2 in tumour-with-matched-normal mode using the gnomAD v3 panel-of-normals and the Broad gnomAD v3 germline resource. Variants were filtered with GATK FilterMutectCalls and additionally required to have a tumour allele frequency ≥ 5%, depth ≥ 20, and ≤ 1/200 reads supporting the alternate allele in the matched normal. Copy-number variants were called with CNVkit (v0.9.10). Microsatellite-instability status was inferred with MSIsensor-pro. Variants were annotated with Ensembl VEP (release *[GRCh38, v110]*) and ranked by predicted clinical impact using OncoKB and the Belgian ComPerMed five-tier framework [Froyen et al., *Cancers* 2019].

S1.4. Variant classification and reporting

Variants were classified into five biological tiers (Tier IA–V) and four clinical tiers (Tier 1–4) per the Belgian ComPerMed framework: Tier IA, FDA-/EMA-approved targets in the same indication; Tier IB, in another indication; Tier IIC, predictive evidence from clinical trials; Tier IID, preclinical or case-report evidence; Tier III, variants of uncertain significance (VUS); Tier IV, benign/likely-benign. Only Tier IA–IID variants were reported as actionable; Tier III variants were tabulated separately as VUS. Pathogenic and likely-pathogenic germline variants in cancer predisposition genes were flagged for referral to the clinical genetics service. The variants reported in the main manuscript (Table 2) are those classified as Tier IA–IID at the time of analysis.

Supplementary Table S1. Antibodies and staining conditions used for immunohistochemistry

Antibody	Clone	Dilution	Antigen retrieval	Source	Positive control
CD3	2GV6	RTU	CC1	Roche (Ventana), Tucson, AZ, USA	Tonsil
CD8	C8/144B	RTU	CC1	Agilent (Dako), Santa Clara, CA, USA	Tonsil
FOXP3	236A/E7	1:50	CC1	Abcam, Cambridge, UK	Tonsil
PD-1	NAT105	RTU	CC1	Roche (Ventana), Tucson, AZ, USA	Tonsil
PD-L1	22C3	1:40	CC1	Agilent (Dako), Santa Clara, CA, USA	Tonsil
PD-L1	SP142	RTU	CC1	Roche (Ventana), Tucson, AZ, USA	Tonsil
PD-L2	366C.9E5	1:50	CC1	Merck Millipore, Burlington, MA, USA	Tonsil
NY-ESO-1	E978	1:50	CC2	Thermo Fisher Scientific, Waltham, MA, USA	Testis

All stainings were performed on the VENTANA BenchMark ULTRA platform (Roche Diagnostics, Tucson, AZ, USA). RTU, ready to use; CC1, cell-conditioning solution 1 (Tris-EDTA-based, pH 8.5); CC2, cell-conditioning solution 2 (citrate-based, pH 6); AR, antigen retrieval. Dako (Agilent) antibodies were used on the VENTANA BenchMark ULTRA platform; cross-platform interchangeability has been validated.

Table S2. Paired pre- and post-NAC genetic alterations, variant-level detail

Table S2. Complete variant-level listing for the 12 paired cases, previously presented as main-text Table 2 and moved to the Supplementary Materials at the reviewers' request. The summary oncoprint is Figure 5 of the main text.

	**Amplifications (TSO-500) pre-NAC**	**Amplifications (TSO-500) post-NAC**	**Variants (TSO-500) pre-NAC**	**Variants (TSO-500) post-NAC**	**Variants (WES) pre-NAC**	**Variants (WES) post-NAC**
Patient 13	None detected	None detected	TP53 R248Q<br>FBXW7 R385G<br>(7 VUS)	TP53 R248Q<br>FBXW7 R385G<br>TSC1 c.2626-2dup<br>(EP300 VUS)	TP53 R248Q<br>FBXW7 R385G<br>TSC1 c.2626-2dup<br>(EP300 VUS)	TP53 R248Q<br>FBXW7 R385G
Patient 17	MYC (3.3)	MYC (2)	TP53 T256Hfs*8<br>FGFR3 K652E<br>FGFR3 I540V<br>PIK3CA H1047R<br>KDM6A splice<br>DNMT3B Q475*<br>(17 VUS)	TP53 T256Hfs*8<br>FGFR3 K652E<br>FGFR3 I540V<br>PIK3CA H1047R<br>KDM6A splice<br>(16 VUS)	TP53 T256Hfs*8<br>FGFR3 K652E<br>FGFR3 I540V<br>KDM6A splice<br>KMT2D C3132Lfs*44<br>(ATM VUS, ERBB3 VUS, KMT2D VUS, MB21D2 VUS)	TP53 T256Hfs*8<br>FGFR3 K652E<br>FGFR3 I540V<br>PIK3CA H1047R<br>KDM6A splice<br>(16 VUS)
Patient 34	None detected	None detected	/	/	TP53 S241F<br>TP53 C238S<br>(ERBB3 VUS, FAT1 VUS, H3C2 VUS, KMT2C VUS)	/
Patient 15	None detected	None detected	/	/	CDKN2A E27*<br>CDKN2A S12*<br>KDM56A K29*<br>TP53 S183*<br>KMT2C Q220*<br>(FGFR3 VUS, 2x FOXA1 VUS, KMT2A VUS, 3x KMT2C VUS, KMT2D VUS)	CDKN2A E27*<br>KDM56A K29*<br>TP53 S183*<br>KMT2C Q220*<br>(KMT2C VUS)
Patient 36	/	/	/	/	/	/
Patient 23	CCNE1 (15.4), EGFR (3.9), MYC (2.3), CHEK1 (1.9)	CCNE1 (8.6), EGFR (4.7)	KDM6A Q687Nfs*56<br>PIK3CA E545K<br>SHQ1 Q551*<br>MUTYH G396D<br>TP53 H168L (VUS)<br>(18 VUS)	KDM6A Q687Nfs*56<br>PIK3CA E545K<br>SHQ1 Q551*<br>MUTYH G396D<br>TP53 H168L (VUS)<br>(18 VUS)	KDM6A Q635Nfs*56<br>PIK3CA E545K<br>TP53 H168L (VUS)<br>(KMT2C VUS, KANSL1 VUS, CREBBP VUS)	KDM6A Q635Nfs*56<br>PIK3CA E545K<br>TP53 H168L (VUS)<br>(KMT2C VUS, KANSL1 VUS, CREBBP VUS)
Patient 42	None detected	None detected	KDM6A R1415*<br>CIC S1932Pfs*9<br>ERCC2 E606Q<br>RB1 E252*<br>RB1 Q850*<br>MITF E318K<br>(25 VUS)		ERCC2 E582Q<br>RB1 E282*<br>RB1 Q850*<br>(ELF3 VUS, ERBB3 VUS, 2x FBXW7 VUS, FOXQ1 VUS, RB1 VUS, RBM10	(KMT2C VUS)

					VUS, SSH3 VUS)	
Patient 37	MET (9.7), CCND1 (5.4)	CCND1 (1.96)	TP53 I255F CDKN2A E88Gfs*32 CDKN2A R58* KDM6A P1449Ifs*142 FAT1 Y3372* RBM10 E494* NFE2L2 E79Q (lage VAF) FAT1 M1533Rfs*9 (lage VAF) FBXW7 S598* (lage VAF) TP53 Q136E (lage VAF) CASP8 R430* (lage VAF) (11 VUS)	TP53 I255F CDKN2A E88Gfs*32 CDKN2A R58* KDM6A P1449Ifs*142 FAT1 Y3372* RBM10 E494* NFE2L2 E79Q FAT1 M1533Rfs*9 (15 VUS)	TP53 I255F CDKN2A E88Gfs*32 CDKN2A R58* KDM6A P1449Ifs*142 FAT1 Y3372* RBM10 E494* NFE2L2 E79Q (lage VAF) FAT1 M1533Rfs*9 (lage VAF) FBXW7 S598* (lage VAF) TP53 Q136E (lage VAF) CASP8 R430* (lage VAF) FOXQ1 L181Cfs*223 (KMT2D VUS)	TP53 I255F CDKN2A E88Gfs*32 CDKN2A R58* KDM6A P1449Ifs*142 FAT1 Y3372* RBM10 E494* NFE2L2 E79Q FAT1 M1533Rfs*9 FOXQ1 L181Cfs*223 (KMT2A VUS, KMT2D VUS)
Patient 41	None detected	None detected	TP53 P151S NOTCH3 D1221Gfs*44 MUTYH G369D (8 VUS)	TP53 P151S NOTCH3 D1221Gfs*44 MUTYH G369D (8 VUS)	TP53 P151S (KMT2C VUS, STAG2 VUS)	TP53 P151S (KMT2C VUS, STAG2 VUS) - veel achtergrondruis - varianten lage VAF
Patient 12	None detected	None detected			ARID1A E1531*, CREBBP Q935*, RB1 Q62*, STAG2 L657Dfs*3 (ARID1A VUS, 3x KMT2C VUS, SSH3 VUS, USP28 VUS)	ARID1A E1531*, CREBBP Q935*, PIK3CA E545K, RB1 Q62*, STAG2 L657Dfs*3 (ARID1A VUS, 2x H3C2 VUS, 3x KMT2C VUS, SSH3 VUS, USP28 VUS)
Patient 22	None detected	None detected	PIK3CA E545K, FH R101* (9 VUS)	PIK3CA E545K, FH R101* (9 VUS)	PIK3CA E545K, FH R101* (9 VUS)	PIK3CA E545K, RHOB E47K (FAT1 Q3959E VUS)
Patient 32	MYC (2), CCND1 (2)	MYC (2.1)	TP53 Q104* NFE2L2 E79Q (13 VUS)	TP53 Q104* NFE2L2 E79Q MDC1 splice? (14 VUS)	TP53 Q104* NFE2L2 E79Q (13 VUS)	TP53 Q104* NFE2L2 E79Q KMT2C A2923Cfs*2 KMT2D H5186Tfs*57 KMT2D Q3796* (ATM VUS, KMT2C VUS, NRAS VUS)

VUS, variant of uncertain significance; "/" indicates that the assay was not performed or returned no result for that specimen. Copy number for amplifications is given in parentheses.

Table S3. Baseline proportions with 95% confidence intervals

Table S3. Exact proportions and 95% Wilson score confidence intervals for the categorical baseline variables in main-text Table 1.

Variable	pCR n/N	pCR %	pCR 95% CI	No pCR n/N	No pCR %	No pCR 95% CI	p
Male sex	9/11	81.8	52.3–94.9	23/31	74.2	56.8–86.3	>0.99
Clinical stage cT3–4	7/11	63.6	35.4–84.8	24/31	77.4	60.2–88.6	0.437
Clinical node positive	3/11	27.3	9.7–56.6	12/31	38.7	23.7–56.2	0.717
Hydronephrosis	1/11	9.1	1.6–37.7	16/31	51.6	34.8–68.0	0.016
Previous intravesical BCG	1/11	9.1	1.6–37.7	4/31	12.9	5.1–28.9	>0.99
Variant histology (any)	1/11	9.1	1.6–37.7	4/31	12.9	5.1–28.9	>0.99
Completed 4 dd-MVAC cycles	10/11	90.9	62.3–98.4	27/31	87.1	71.1–94.9	>0.99

Confidence intervals are Wilson score intervals, which retain nominal coverage at these sample sizes where the Wald interval does not. p-values are Fisher exact, two-sided.

Table S4. Baseline immune markers by pathologic response

Table S4. Baseline (TURBT) immune-marker values compared between patients who did and did not achieve a pathologic complete response, with Benjamini–Hochberg adjusted p-values.

Marker	n (pCR)	Median (pCR)	n (no pCR)	Median (no pCR)	Effect size r	p	q
CD3 (grade)	7	2.0	18	2.0	+0.03	0.915	0.915
CD3 score	7	3.0	18	3.0	+0.16	0.537	0.914
CD8 (grade)	7	2.0	18	1.0	+0.13	0.600	0.914
CD8 score	7	2.0	18	2.0	+0.10	0.686	0.914
FOXP3 (grade)	7	1.0	18	1.0	+0.05	0.828	0.915
PD-1 (%)	7	0.0	18	0.0	+0.10	0.602	0.914
PD-L2 (%)	7	0.0	18	0.5	-0.19	0.443	0.914
NY-ESO-1 (grade)	6	0.5	18	0.5	-0.14	0.612	0.914

Mann–Whitney U test; effect size is the rank-biserial correlation. No marker was associated with pathologic response before or after correction for multiple comparisons. With only seven evaluable responders these comparisons are severely underpowered and should be interpreted as non-informative rather than as evidence of absence.

Table S5. Analysis populations and missing data

Table S5. Definition of each analysis population, the number of patients contributing, and the reasons for non-contribution.

Analysis	n contributing	Denominator	Reason for non-contribution
Baseline characteristics	54	54	None — complete cohort
Pathologic response (pCR vs residual)	42	54	12 did not undergo cystectomy (progressive disease 4, TURB alone 4, primary chemoradiotherapy 2, primary radiotherapy 1, upper-tract carcinoma 1)
Overall and recurrence-free survival	54	54	None; response-stratified comparisons restricted to the 42 operated patients
Cox proportional-hazards models	42	54	Restricted to operated patients
Immunohistochemistry, baseline	31	54	23 with no evaluable TURBT block or insufficient tissue
Immunohistochemistry, paired	22	54	12 no cystectomy specimen; 20 without evaluable paired material
Baseline marker vs pCR	25	42	17 operated patients without an evaluable baseline specimen
Paired genomic profiling (alterations)	12	54	12 no cystectomy specimen; 11 pCR with no residual tumour; 18 insufficient or degraded DNA; 1 excluded at quality control
Fully evaluable at both timepoints	10	12	1 post-NAC specimen failed sequencing; 1 case failed at both timepoints
TMB, paired WES	10	54	As above; WES not evaluable in remaining cases
TMB, paired TSO-500	7	54	TSO-500 performed on a subset of the paired cases
Cross-assay TMB comparison	8	54	Both assays available at baseline in 8 patients

The paired molecular populations are not a random sample of the cohort: patients achieving a pathologic complete response are excluded by definition, because no residual tumour remains to sequence. The molecular results therefore describe chemoresistant disease.

Table S6. Sensitivity analysis using the averaged field score

Table S6. Paired changes in immune markers analysed with the composite averaged field score rather than the categorical grade reported in the main text. The score follows the approach of De Meulenaere et al. [39,40] but has not been validated as a measurement instrument in urothelial carcinoma; it is shown here to demonstrate that the choice of scale does not alter the conclusions. An averaged score was recorded at both timepoints only for CD3 and CD8.

Marker	Paired n	Pre-NAC median (IQR)	Post-NAC median (IQR)	Effect size r	p	q
CD3 score	21	3.0 (2.0–3.0)	2.0 (2.0–3.0)	-0.62	0.053	0.106
CD8 score	20	2.0 (1.8–2.0)	1.0 (1.0–2.0)	-0.43	0.117	0.117

Wilcoxon signed-rank test; q = Benjamini–Hochberg adjusted p-value. The direction of change and the significance of CD3 are unchanged from the grade-based analysis in main-text Table 3, supporting the robustness of the reported findings to the choice of measurement scale.

Supplementary Methods S2. Immunohistochemistry scoring — detailed criteria

S2.1. Grading scale

Immune infiltration was graded on a four-level semi-quantitative scale applied to three randomly chosen fields at ×20 objective magnification per slide: none, no positive cells; low, sporadic positive cells; moderate, moderate numbers of positive cells; strong, abundant positive cells. An overall grade was assigned per marker and specimen and coded 0–3 for analysis. CD3, CD8, FOXP3 and NY-ESO-1 were graded on this scale; PD-1 and PD-L2 were recorded as the percentage of positive cells.

S2.2. Criteria for classifying a specimen as impossible to evaluate

A specimen was classified as impossible to evaluate, and excluded from the analysis of that marker, if any of the following applied: (1) virtually no tumour stroma was present in the section; (2) only a minimal invasive carcinoma component was present, most often in superficial TURBT fragments; or (3) tumour-associated immune infiltrate could not be reliably distinguished from pre-existing lymphoid aggregates or from the dense chronic inflammation that follows intravesical therapy or recent instrumentation. These criteria, rather than tissue exhaustion alone, account for the difference between the 54 patients in the cohort and the 31 with an evaluable baseline specimen (Figure 1, Table S5).

S2.3. Composite numerical score

In addition to the categorical grade, a composite numerical score was recorded by averaging the individual field scores, as in our earlier work in oropharyngeal squamous cell carcinoma [39,40]. This composite score has not been validated as a measurement instrument in urothelial carcinoma, and the categorical grade is therefore used throughout the manuscript. A sensitivity analysis based on the averaged score is presented in Table S6; the direction and significance of the reported changes are unaffected by the choice of scale.

S2.4. Observer agreement

All slides were scored independently by a uropathologist (S.V.) and the first author (D.D.M.), both blinded to pathologic response, clinical outcome and specimen timepoint. Discordant scores were re-reviewed jointly on a double-headed microscope and resolved by consensus. Only consensus scores were retained in the study database, so a formal inter-observer agreement statistic could not be computed retrospectively. Discordance was in all cases limited to adjacent categories and never exceeded one grade.

Table S7. Molecular data availability per paired case

Table S7. Sequencing results available at each timepoint for the 12 patients in whom paired genomic analysis was attempted. Patient numbers correspond to Figure 2. Eleven patients yielded a result on the pre-treatment (TURBT) specimen and ten at both timepoints; one patient (12) failed sequencing at both timepoints and one (22) at the post-treatment timepoint only.

Patient	WES TMB pre	WES TMB post	TSO-500 TMB pre	TSO-500 TMB post	Both timepoints
12	—	—	—	—	no
13	17	21	8.1	6.5	yes
15	29	20	24.6	—	yes
17	24	25	15.2	14.3	yes
22	24	—	—	—	no
23	39	45	—	—	yes
32	22	14	3.2	2.4	yes
34	29	25	—	—	yes
36	25	25	8.9	11.1	yes
37	18	41	3.2	4.8	yes
41	20	18	7.1	6.3	yes
42	21	26	7.1	10.3	yes

TMB is given in mutations per megabase. "—" indicates that the assay returned no result for that specimen. Patient 12 had DNA extracted at both timepoints but no sequencing result was obtained; patient 22 failed at the post-treatment timepoint.

Table S8. Patients and events per covariate category

Table S8. Number of patients and observed events in each covariate category of the Cox proportional-hazards models, restricted to the 42 operated patients (11 deaths, 17 recurrence events). These counts explain the width of the confidence intervals in main-text Table 2: several categories contain very few events.

Covariate	Category	Patients	OS events	RFS events
Pathologic response	Residual disease	31	10	16
Pathologic response	pCR	11	1	1
Hydronephrosis	No	25	7	8
Hydronephrosis	Yes	17	4	9
Clinical nodal status	cN+	15	5	8
Clinical nodal status	cN0	25	6	9
Clinical T stage	cT2	5	2	2
Clinical T stage	cT3-4	31	9	13
Completed 4 cycles	No	5	1	2
Completed 4 cycles	Yes	37	10	15

OS, overall survival; RFS, recurrence-free survival. Clinical T stage was not assessable in 6 patients and clinical nodal status in 2, so those categories do not sum to 42. Only pathologic response and clinical nodal status entered the multivariable models, together with hydronephrosis.
